# Publisher Correction: Observation of dark states in a superconductor diamond quantum hybrid system

**DOI:** 10.1038/ncomms16202

**Published:** 2018-03-29

**Authors:** Xiaobo Zhu, Yuichiro Matsuzaki, Robert Amsüss, Kosuke Kakuyanagi, Takaaki Shimo-Oka, Norikazu Mizuochi, Kae Nemoto, Kouichi Semba, William J. Munro, Shiro Saito

Nature Communications 5: Article number: 3524; DOI: 10.1038/ncomms4524 (2014); Published 04 08 2014; Updated 03 29 2018

The original HTML version of this Article had an incorrect article number of 3424; it should have been 3524. This has now been corrected in the HTML; the PDF version of the Article was correct from the time of publication.

